# Taking Multiple Infections of Cells and Recombination into Account Leads to Small Within-Host Effective-Population-Size Estimates of HIV-1

**DOI:** 10.1371/journal.pone.0014531

**Published:** 2011-01-13

**Authors:** Rajesh Balagam, Vasantika Singh, Aparna Raju Sagi, Narendra M. Dixit

**Affiliations:** 1 Department of Chemical Engineering, Indian Institute of Science, Bangalore, India; 2 Bioinformatics Centre, Indian Institute of Science, Bangalore, India; BC Centre for Excellence in HIV/AIDS, Canada

## Abstract

Whether HIV-1 evolution in infected individuals is dominated by deterministic or stochastic effects remains unclear because current estimates of the effective population size of HIV-1 *in vivo*, *N_e_*, are widely varying. Models assuming HIV-1 evolution to be neutral estimate *N_e_*∼10^2^–10^4^, smaller than the inverse mutation rate of HIV-1 (∼10^5^), implying the predominance of stochastic forces. In contrast, a model that includes selection estimates *N_e_*>10^5^, suggesting that deterministic forces would hold sway. The consequent uncertainty in the nature of HIV-1 evolution compromises our ability to describe disease progression and outcomes of therapy. We perform detailed bit-string simulations of viral evolution that consider large genome lengths and incorporate the key evolutionary processes underlying the genomic diversification of HIV-1 in infected individuals, namely, mutation, multiple infections of cells, recombination, selection, and epistatic interactions between multiple loci. Our simulations describe quantitatively the evolution of HIV-1 diversity and divergence in patients. From comparisons of our simulations with patient data, we estimate *N_e_*∼10^3^–10^4^, implying predominantly stochastic evolution. Interestingly, we find that *N_e_* and the viral generation time are correlated with the disease progression time, presenting a route to *a priori* prediction of disease progression in patients. Further, we show that the previous estimate of *N_e_*>10^5^ reduces as the frequencies of multiple infections of cells and recombination assumed increase. Our simulations with *N_e_*∼10^3^–10^4^ may be employed to estimate markers of disease progression and outcomes of therapy that depend on the evolution of viral diversity and divergence.

## Introduction

The within-host genomic evolution of HIV-1 is driven by both deterministic forces such as selection and stochastic forces such as random genetic drift. The large census population of HIV-1 infected cells, ∼10^7^–10^8^ in a typical patient [1], suggests the predominance of deterministic forces underlying HIV-1 evolution. Yet, inter-patient variations in the duration of the asymptomatic phase of infection, in the set point viral load, and in the rates and patterns of the emergence of drug resistant and immune escape mutants are large [2]. For instance, genotypic resistance to the protease inhibitor ritonavir arose at widely different times and through distinct combinations of mutations in different patients [3]. One reason for these inter-patient variations may be variations in host-genetic factors [2], [4]. Recently, variation in the copy number of the CCL3L1 gene, a potent suppressor of HIV-1, was shown to be associated with the variation in the susceptibility of individuals to HIV-1 [5]. Inter-patient variations may also arise if stochastic forces underlying HIV-1 evolution dominate deterministic forces. The relative influence of stochastic forces is determined by the within-host effective population size, *N_e_*: despite the large census population, stochastic forces may dominate if *N_e_* is small [6]. Current estimates of *N_e_* are widely varying, ranging from a few hundred to >10^5^ cells, which leaves unclear the nature of HIV-1 evolution *in vivo* and limits our ability to describe disease progression and outcomes of therapy [7].

*N_e_* is defined as the size of an idealized population that has the same population genetic properties as that of the natural population [7], [8]. To estimate *N_e_*, a calibration quantity that is measurable in the natural population, such as genomic diversity, is predicted using a model of the evolution of an idealized population, such as the Wright-Fisher model. The calibration quantity is a function of the population size, *C*, in the idealized model. *N_e_* is then identified as that value of *C* at which the predicted value of the calibration quantity matches the value measured in the natural population. Accurate estimation of *N_e_* relies on an idealized model that closely mimics the evolution of the calibration quantity in the natural population [7]. The idealized model with *C* = *N_e_* may then be employed to predict other quantities that describe the behaviour of the natural population but are difficult to measure so long as the evolutionary forces that govern the latter quantities are the same as those underlying the calibration quantity and incorporated in the idealized model [7].

To estimate *N_e_* for HIV-1 *in vivo*, several studies have employed idealized models that assume HIV-1 evolution to be neutral [3], [9]–[13]; *i.e.*, that genomic variations do not lead to variation in fitness and therefore selective forces are inconsequential. By comparisons of model predictions with data on polymorphisms in the *env* or the *gag-pol* region of HIV-1, the latter studies obtained *N_e_*∼10^2^–10^4^. These latter studies employed several tests to ascertain the predominant neutrality of HIV-1 evolution. More recent evidence, however, points to significant selective pressures on both the *env* and the *gag-pol* regions [7], [12], [14], rendering uncertain the estimates of *N_e_* obtained by neutral models. Rouzine and Coffin considered HIV-1 evolution with selection and predicted the frequency of the least abundant haplotype in a two-locus/two-allele model [15]. By comparison with data from *env* and *pro* regions, the latter model yielded *N_e_*>10^5^. The latter model, however, did not include recombination. Growing evidence [16], [17], including the observation of circulating recombinant forms of HIV-1 as well as recombinant forms unique to individuals [18], points to the significance of recombination in the evolution of HIV-1. Recombination alters the association of mutations and influences the prevalence of haplotypes [19]-[22], which in turn may affect the estimate of *N_e_* obtained by Rouzine and Coffin. It is of importance therefore to estimate *N_e_* using a model of HIV-1 evolution that incorporates both selection and recombination.

Substantial efforts are ongoing to describe HIV-1 evolution in the presence of recombination [23]-[35]. Recent advances in mathematical modelling and stochastic simulations have provided valuable insights into the role of recombination in the genomic diversification of HIV-1 *in vivo*, particularly in the context of the development of resistance to antiretroviral therapy (reviewed in [22]). Specifically, the influence of recombination is predicted to depend sensitively on *N_e_* and on the nature of fitness interactions between loci, characterized by epistasis: When *N_e_* is small, recombination tends to lower viral genomic diversity independent of epistasis, whereas when *N_e_* is large, recombination lowers (enhances) diversity if epistasis is positive (negative). Further, recombination is also predicted to lower the waiting time for the emergence of viral genomes carrying new, potentially favourable combinations of mutations.

Our aim is to employ a model of HIV-1 evolution that accurately mimics viral genomic diversification in infected individuals as a function of the population size and estimate *N_e_* from comparisons of model predictions with patient data. Analytical models of HIV-1 evolution with recombination allow description of viral evolution not only at the extremes of very small and very large *N_e_*, where drift and selection, respectively, dominate, but also at intermediate values of *N_e_* where both selection and drift remain important simultaneously [24], [27], [28], [31], [33]-[35]. The models, however, are restricted to a small number of loci and/or to simple (multiplicative) fitness landscapes. Experimental data on viral diversification, in contrast, is available over genomic regions that are up to several hundred nucleotides long (e.g., see [36]). Besides, the best available description of the HIV-1 fitness landscape [37] points to significant deviations from a simple multiplicative fitness profile. To overcome these limitations of analytical models, we have recently developed bit-string simulations of the within-host genomic diversification of HIV-1 [32]. Our simulations consider large genome lengths and incorporate mutation, infection of cells by multiple virions, recombination, fitness selection, and epistatic interactions between multiple loci, thereby presenting a detailed description of the evolution of viral diversity and divergence in infected individuals [32]. In particular, our simulations elucidate the role of recombination in HIV-1 diversification as a function of *N_e_* and with the experimentally determined fitness landscape. Here, we apply the simulations to describe patient data and obtain estimates of *N_e_*. We examine the dependence of our estimates of *N_e_* on the frequency of multiple infections of cells and on the nature of the fitness landscape, which remain to be established *in vivo*. Finally, we revisit the large estimate of *N_e_* obtained by Rouzine and Coffin [15] by incorporating multiple infections of cells and recombination in their two-locus/two-allele model.

## Results

### Simulations of the within-host genomic diversification of HIV-1

We perform simulations to predict the evolution of viral diversity, *d_G_*, and divergence, *d_S_*, in an HIV-1 infected individual (Methods). Diversity is a measure of the genomic variation in the viral population at any given time, whereas divergence is a measure of the deviation of the viral genomes from the founder strain. Simulations begin with the synchronous infection of a fixed population, *C*, of cells by identical homozygous virions. Following infection, viral RNA are reverse transcribed to proviral DNA, during which process mutation and recombination introduce genomic variation. Proviral DNA are then transcribed into viral RNA, which are randomly assorted into pairs and released as new virions. New virions are chosen according to their fitness to infect the next generation of uninfected cells, and the cycle is repeated.

We employ parameter values representative of HIV-1 infection *in vivo*. We consider a viral genome length of *L* = 100 nucleotides, which spans the experimental fitness landscape [37] (Methods) and captures expected epistatic interactions between multiple loci and is also similar to the genome lengths examined in the experiments we consider [36] (see below). We assume that mutations occur independently at each of the *L* sites at the rate *µ* = 3×10^−5^ substitutions per site per replication [38]. Because a majority of HIV mutations are transitions [38], we ignore transversions, insertions and deletions. Following recent estimates, we choose the recombination rate *ρ* = 8.3×10^−4^ crossovers per site per replication [17], [31]. The relative fitness of the founder strain, determined by ξ (Methods), remains unknown. Recent studies show that the founder strain evolves under selective forces and is distinct from the strain(s) dominant in the chronic infection phase [39]–[41]. In a previous study, we found that ξ∼0.05–0.1 provides best fits to the evolution of divergence and diversity in the two patients examined [32]. This is in accordance with the maximum divergence of ∼0.1 in the patient data we consider [36]. Here, we therefore set ξ = 0.1. We let selection follow the fitness landscape determined experimentally [37] (Methods). We also examine the effects of alternative (multiplicative) fitness landscapes on our estimates of *N_e_*. The frequency of multiple infections of cells *in vivo* remains uncertain. Infections of individual cells by multiple virions allow the formation of heterozygous progeny virions and set the stage for recombination to introduce genomic variation [42]. Jung et al. [16] found that infected splenocytes in the two patients they examined harbored between 1 and 8 proviruses with a mean of 3–4 proviruses per cell. In contrast, Josefsson et al. [43] recently observed that a vast majority of the peripheral blood mononuclear cells in four patients harbored single proviruses. Here, we therefore perform simulations with both these patterns of multiple infections of cells: We first follow Jung et al. [16] and let each cell be infected by *M* = 3 virions. We then repeat our simulations with *M* drawn from a distribution that follows from a model of viral dynamics (Methods) and that mimics the observations of Josefsson et al. [43]. Although the viral burst size is large, ∼10^2^–10^4^ [1], [44], [45] only a few (2–3 per cell [46]) of the virions produced may be infectious [46]–[48]. More recent estimates of the basic reproductive ratio of HIV-1 *in vivo* suggest the production of 6–8 infectious virions per cell [49]. Here, we let each cell produce *P* = 5 infectious progeny virions [32]. We let simulations proceed to 4000 generations (∼10–12 years).

### Time-evolution of viral diversity and divergence

**Figure 1:**
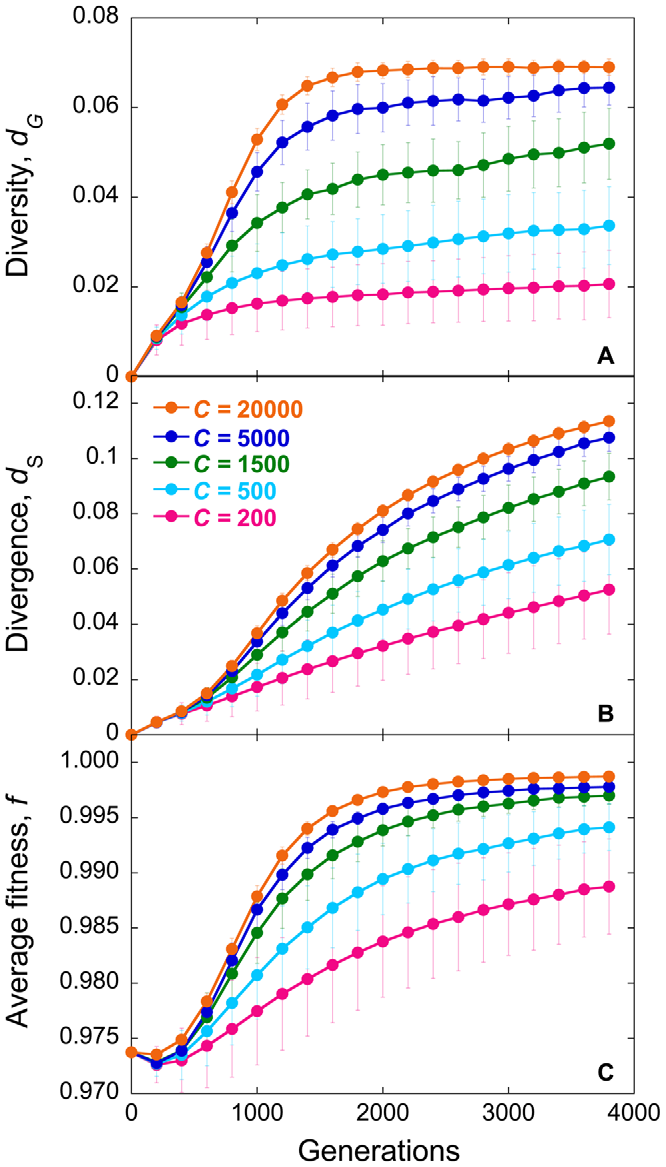
Simulations of viral genomic diversification. The evolution of (A) viral diversity, *d_G_*, (B) divergence, *d_S_*, and (C) average fitness, *f*, with generations predicted by our simulations for different population sizes, *C*. Each cell is assumed to be infected with *M* = 3 virions. Error bars represent standard deviations.

In Fig. 1, we present the evolution of *d_G_* and *d_S_*, and of the mean viral fitness, *f*, with the number of generations for different values of the population size, *C*. We employ *M* = 3 and the experimental fitness landscape (Methods). Initially, *d_G_* and *d_S_* are zero because infection begins with identical genomes. As infection progresses, mutations accumulate and both *d_G_* and *d_S_* rise. Recombination may also accelerate the accumulation of mutations and enhance viral diversification (see below) [32]. Mutations typically incur a fitness penalty. Mutant genomes may thus be lost due to selection. Selection drives evolution towards the fittest sequence. *f* consequently rises. At the same time, genomes may be lost due to random genetic drift. Eventually, a balance between mutation and recombination, which enhance viral diversification, and selection and drift, which constrain viral diversification, is reached; accordingly, *d_G_*, *d_S_*, and *f* attain equilibrium values.

In a previous study, we have examined in detail the influence of variations in parameter values on the evolution of *d_G_*, *d_S_*, and *f* [32]. We found that mutations alone result in an equilibrium with *d_G_* = *d_S_* = 0.5, which follows from a balance between forward and back mutations, both assumed to be equally likely. An increase in the frequency of multiple infections of cells, *M*, resulted in an increase in *d_G_* because more diverse genomes formed proviruses as *M* increased, effectively lowering drift. *d_S_*, however, was unaffected by variations in *M*. Selection lowered *d_G_* as genomes close to the fittest sequence were increasingly favored. Further, when the fittest sequence was also the founder sequence, selection also lowered *d_S_*, as selection then limited diversification from the founder sequence. The influence of recombination was dependent on the nature of epistatic interactions between loci and on the population size, *C*. When *C* was small, recombination increased *d_G_*, whereas when *C* was large, recombination lowered *d_G_*. In both cases, however, recombination enhanced *f*. This influence of recombination is consistent with current population genetics theories [19]–[21]: When *C* is small, random genetic drift creates negative linkage disequilibrium according to the Hill-Robertson effect [50]. Recombination then lowers the magnitude of this linkage disequilibrium and enhances viral diversity. When *C* is large, linkage disequilibrium has the same sign as that of epistasis [51]. The fitness landscape we considered has a mean positive epistasis [37]. Recombination is then expected to lower the magnitude of the resulting positive linkage disequilibrium and cause a decrease of viral diversity. We recognize though that the influence of recombination depends not only on the mean but also the distribution of epistasis values [52].

Of importance here is that *d_G_* and *d_S_* are sensitive to *C*. We find that *d_G_*, *d_S_*, and *f* increase with *C* (Fig. 1). As *C* increases, the influence of drift diminishes allowing greater viral diversification. Accordingly, *d_G_* increases with *C*. At the same time, the influence of selection increases resulting in higher values of *f*. Stronger selection results in greater evolution towards the fittest sequence and, in this case, away from the founder sequence. Consequently, *d_S_* also increases with *C*. This sensitivity of *d_G_* and *d_S_* to *C* allows estimation of *N_e_* by comparison of our simulations with data from patients.

### Estimation of *N_e_* from comparisons with patient data

In one of the most comprehensive longitudinal studies, Shankarappa et al. determined the evolution of diversity and divergence of the C2-V5 region of the *env* gene over a period of 6–12 years post seroconversion in nine patients [36]. We compare our predictions with their data reported as the mean pairwise distance between viral DNA sequences determined using either a Kimura 2-parameter model or a general time-reversible model with site-to-site variation in substitution rates, both methods yielding similar results. The Kimura 2-parameter distance reduces to the Hamming distance when distances are small [53], as is observed with the data here, allowing us to make direct comparisons of our simulation results with the data.

**Figure 2:**
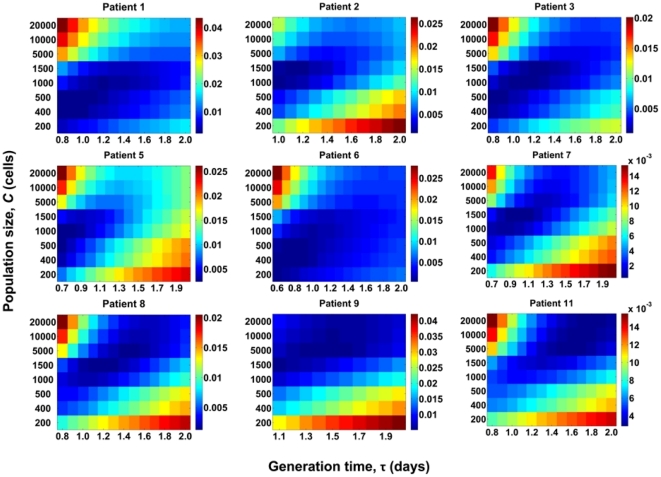
Estimation of *N_e_* from comparisons with data from patients. Sum of squares of the errors (SSE) between data from patients [36] and our predictions of viral diversity, *d_G_*, and divergence, *d_S_*, for different values of the population size, *C*, (Fig. 1) and the viral generation time, τ, shown for each of the nine patients. *C* and τ that yield the lowest SSE provide the best fit to the data. The best-fit value of *C* yields *N_e_* (Table 1).

**Figure 3:**
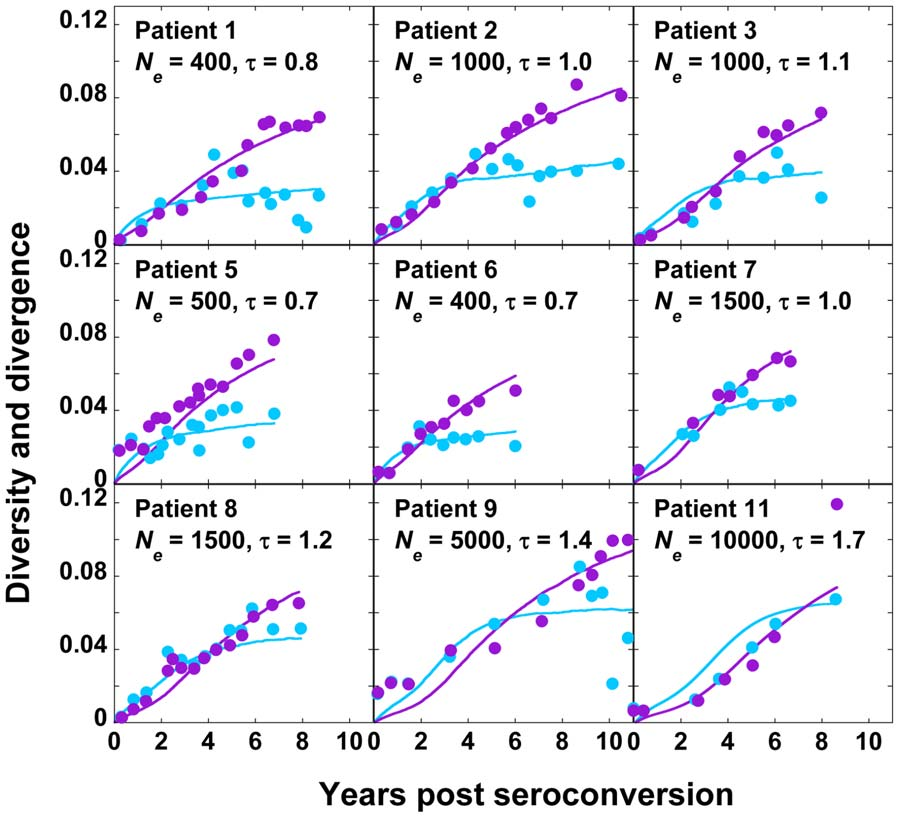
Fits of our simulations to data from patients. Best-fit predictions of our simulations (solid lines) presented with experimental data (symbols) of the evolution of viral diversity, *d_G_*, (cyan) and divergence, *d_S_*, (purple) for each patient. Each cell is assumed to be infected with *M* = 3 virions in our simulations. The values of *N_e_* (cells) and τ (days) employed for the predictions are indicated.

With data from each patient, we compare our predictions of *d_G_* and *d_S_* for different values of *C*, *viz*., 200, 400, 500, 1000, 1500, 5000, 10000, and 20000 cells, and a range of values of the viral generation time, τ, *viz*., 0.6 to 2.0 days per replication [10], [11], [54], [55] in increments of 0.1 days per replication. τ may vary substantially across patients [10], [11], [14], [54], [55]. For each patient, we find the sum of squares of the errors (SSE) between experimental data and our predictions of *d_G_* and *d_S_* for different values of *C* and τ (Fig. 2). The combination of *C* and τ that results in the lowest SSE for a patient yields the best fit of our predictions to data from that patient. The best-fit predictions are shown in Fig. 3. Our simulations provide good fits to data from each of the nine patients. The best fit values of *C* yield *N_e_* (Table 1). We thus find that the mean *N_e_* is ∼2400 (range 400–10000) for these patients. The mean τ is 1.1 day (range 0.7–1.7 day).

**Table 1:** Best-fit parameter estimates and the disease progression time.

Patient	Effective population size, *N_e_* (cells) (Fig. 3)	Viral generation time, τ (days) (Fig. 3)	Effective population size, *N_e_* (cells) (Fig. 7)	Viral generation time, τ (days) (Fig. 7)	Disease progression time (months) [36]
1	400	0.8	1500	1.0	78
2	1000	1.0	5000	1.2	96
3	1000	1.1	5000	1.3	84
5	500	0.7	1500	0.7	72
6	400	0.7	1500	0.9	60
7	1500	1.0	5000	1.0	78
8	1500	1.2	10000	1.4	72
9	5000	1.4	10000	1.3	132
11	10000	1.7	100000	1.8	144
**Mean**	**2367**	**1.1**	**15500**	**1.2**	**91**

Best-fit estimates of *N_e_* and τ obtained by comparison of our simulations with data of viral diversity and divergence from different patients [36] (Figs. 3 and 7). Also listed are the disease progression times determined experimentally [36].

### Correlation with disease progression

**Figure 4:**
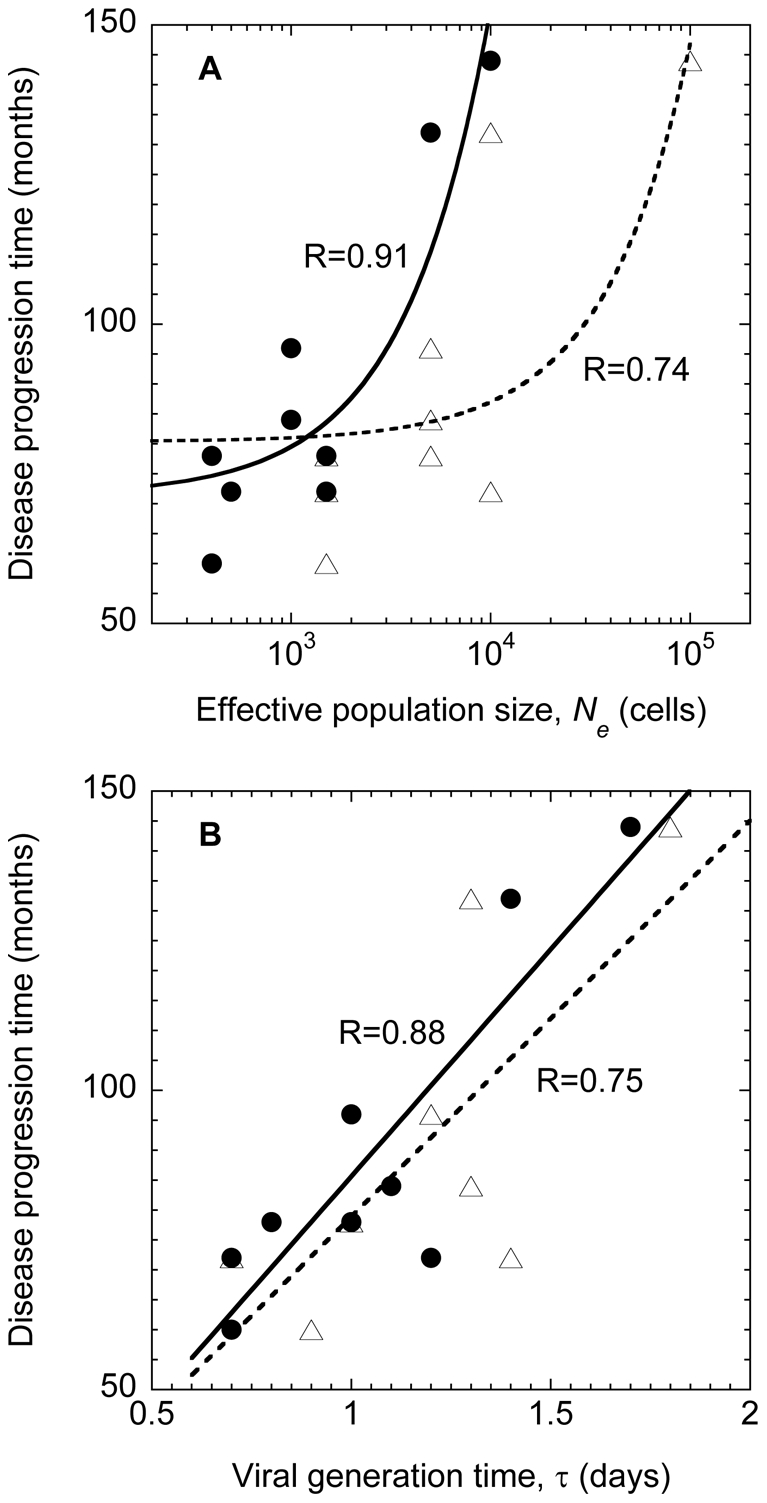
Correlations with disease progression. Correlation of (A) *N_e_* and (B) τ with the disease progression time, or the time from seroconversion for the CD4^+^ T cell count to fall to 200 cells/µL [36]. Symbols represent data obtained from our simulations with the frequency of multiple infections, *M*,  = 3 (circles) and drawn from a distribution based on a viral dynamics model (triangles) (see text). Linear fits (lines) to the data yield Pearson correlation coefficients of (A) 0.91 (circles) and 0.74 (triangles) and (B) 0.88 (circles) and 0.75 (triangles). Note that the *x*-axis in (A) is plotted on a logarithmic scale.

Remarkably, we find that both *N_e_* and τ are strongly correlated with the disease progression time, i.e., the time it takes following seroconversion for the CD4^+^ T cell count to drop below 200 cells/µL (Fig. 4). (Pearson correlation coefficients are 0.91 and 0.88, respectively.) Thus, a small *N_e_* and/or a small τ would imply rapid disease progression. Of the nine patients considered here, seven were typical progressors and two (Patients 9 and 11) were initial non-progressors [36]. Indeed, we find that both *N_e_* and τ are the highest for the latter two patients (Table 1). A small τ would imply fast replication and hence rapid disease progression. The origin of the correlation between *N_e_* and the progression time remains unclear. Nonetheless, the strong correlations we observe present a route to *a priori* estimation of the disease progression time in patients.

### Influence of the frequency of multiple infections of cells

**Figure 5:**
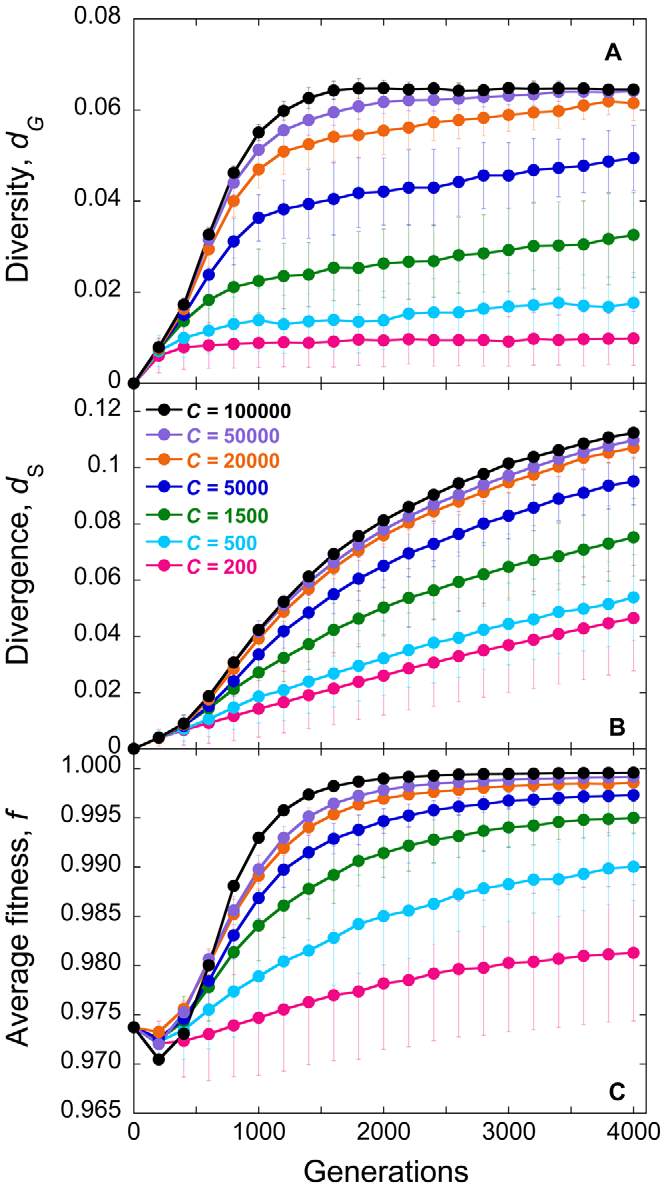
Simulations of viral genomic diversification with a low frequency of multiple infections. The evolution of (A) viral diversity, *d_G_*, (B) divergence, *d_S_*, and (C) average fitness, *f*, with generations predicted by our simulations for different population sizes, *C*. Each cell is assumed to be infected with *M* virions drawn from a distribution based on a viral dynamics model (see text). Error bars represent standard deviations.

**Figure 6:**
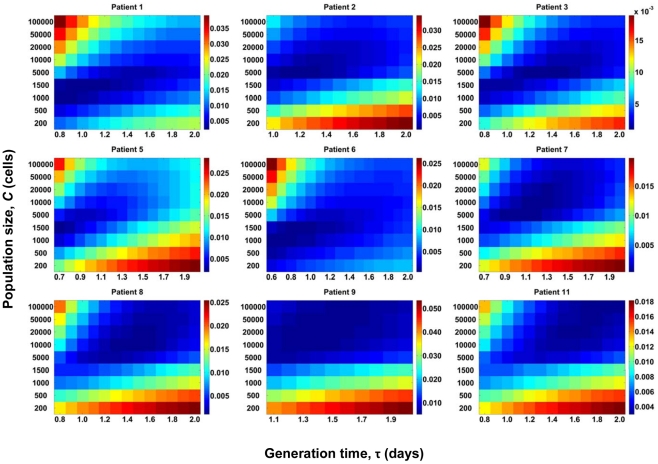
Estimation of *N_e_* from comparisons with data from patients. Sum of squares of the errors (SSE) between data from patients [36] and our predictions of viral diversity, *d_G_*, and divergence, *d_S_*, for different values of the population size, *C*, (Fig. 5) and the viral generation time, τ, shown for each of the nine patients. *C* and τ that yield the lowest SSE provide the best fit to the data. The best-fit value of *C* yields *N_e_* (Table 1).

**Figure 7:**
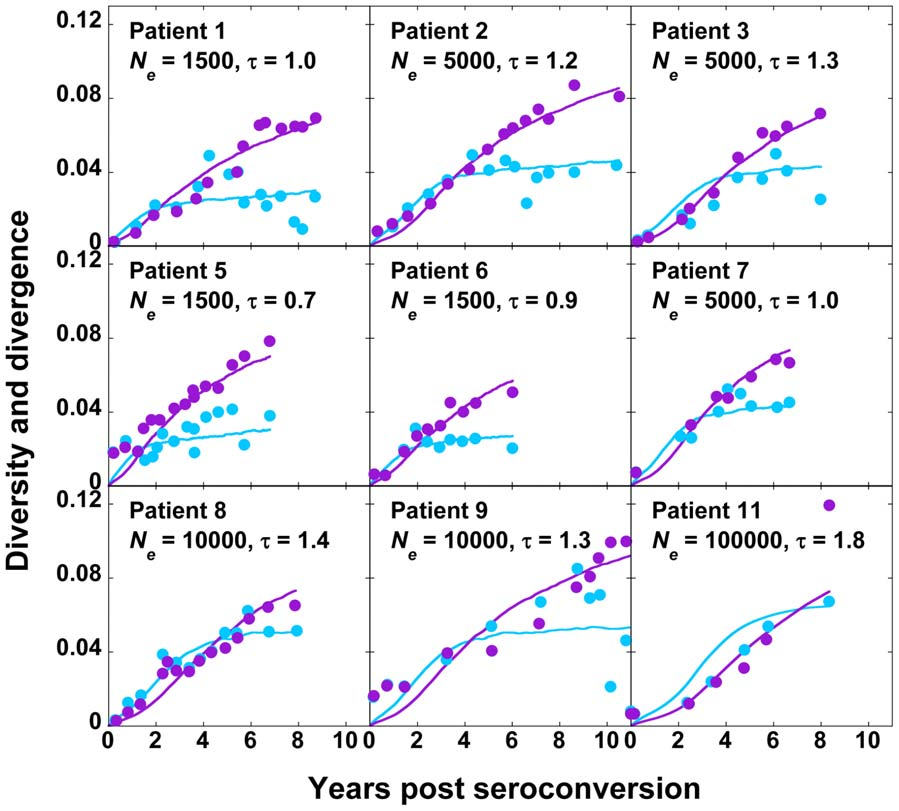
Fits of our simulations to data from patients. Best-fit predictions of our simulations (solid lines) presented with experimental data (symbols) of the evolution of viral diversity, *d_G_*, (cyan) and divergence, *d_S_*, (purple) for each patient. Each cell is assumed to be infected with *M* virions drawn from a distribution based on a viral dynamics model (see text). The values of *N_e_* (cells) and τ (days) employed for the predictions are indicated.

We next examine the effect of a smaller frequency of multiple infections, where *M* is drawn from a distribution based on a model of viral dynamics (Methods) and that is similar to the observations of Josefsson et al. [43]. Thus, ∼77% of the cells are singly infected, ∼19% are doubly infected, and ∼4% are triply infected. In Fig. 5, we present the resulting evolution of *d_G_*, *d_S_*, and *f* for different values of *C*. We find the trends to be similar to those in Fig. 1, except that both *d_G_* and *d_S_* are smaller for any given value of *C* compared to those in Fig. 1. Accordingly, we expect *N_e_* to be higher. In Fig. 6, we present the sum of squares of the errors (SSE) between patient data and our predictions of *d_G_* and *d_S_* for different values of *C* and τ, where we vary *C* from 200 to 100000 cells and τ from 0.6 to 2.0 days per replication. The resulting best-fits are presented in Fig. 7 and estimates of *N_e_* and τ in Table 1. We find that the mean *N_e_* is ∼15500 (range 1500–100000), higher than the value obtained with *M* = 3. The mean τ is 1.2 day (range 0.7–1.8 day), close to the value obtained with *M* = 3. We note that although *N_e_* is higher for each patient compared to the corresponding estimates obtained with *M* = 3, the values of *N_e_* are all in the range 10^3^–10^4^ except for one patient (Patient 11) for whom *N_e_* = 10^5^, indicating the predominance of stochastic forces underlying HIV-1 evolution. Again, we find that both *N_e_* and τ are correlated with the disease progression time, although the correlations are weaker than with *M* = 3 (Fig. 4).

### Influence of the fitness landscape

**Figure 8:**
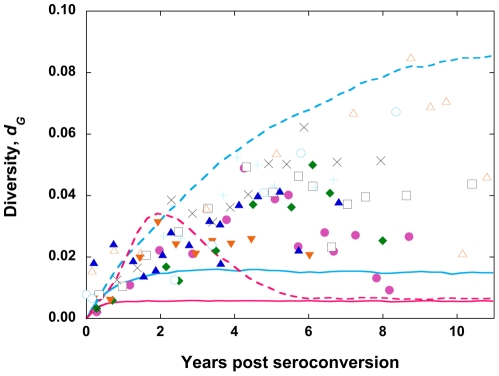
Simulations of viral genomic diversification with a multiplicative fitness landscape and comparisons with patient data. The evolution of viral diversity, *d_G_*, with generations predicted by our simulations (lines) for different population sizes *C* = 200 (solid) and 10000 (dashed) with a multiplicative fitness landscape (see text) with *s* = 0.01 (pink) and 0.001 (cyan). Each cell is assumed to be infected with *M* virions drawn from a distribution based on a viral dynamics model (see text). Different symbols are data from nine different patients [36] shown also in Figs. 3 and 7.

We examine next whether the nature of the fitness landscape has any influence on our estimates of *N_e_*. In the absence of information on the fitness landscape *in vivo*, a multiplicative fitness landscape, which assumes that *f_i_* = exp(-*sd_iF_L*), has been employed in recent studies [28], [30], [35], where *d_iF_* is the normalized Hamming distance of sequence *i* from the fittest sequence and *s* is the fitness penalty per mutation. Here, we perform calculations with the latter landscape for two values of *s*, *viz*., 0.01 and 0.001 (see [35]). *M* is drawn from a distribution based on a model of viral dynamics mentioned above. We find that with *s* = 0.01, both *d_G_* and *d_S_* assume equilibrium values of ∼0.01 over a wide range of values of *C*, which is inconsistent with patient data (Fig. 8). With *s* = 0.001, the evolution of *d_G_* and *d_S_* is consistent with patient data over the range *C* = 200–10000 (Fig. 8). Thus, the resulting values of *N_e_* again lie in the range of 200–10000. The nature of the fitness landscape thus does not appear to influence our estimates of *N_e_* substantially.

### Estimation of *N_e_* using the linkage disequilibrium test: the two-locus/two-allele model

Our estimates of *N_e_* above are consistently smaller than those obtained by Rouzine and Coffin [15]. Here, we examine whether including multiple infections of cells and recombination in the simulations of Rouzine and Coffin alters the resulting estimates of *N_e_*.

Rouzine and Coffin argue that deep in the stochastic regime, one of the four haplotypes in a two-locus/two-allele model is expected to be underrepresented because of the strong influence of drift. As *C* increases, the influence of drift weakens and the frequency of the least abundant haplotype (*lhf*) increases. Comparison of model predictions of *lhf* versus *C* with experimental observations then yields an estimate of *N_e_*. We apply our simulations to predict *lhf* in a two-locus/two-allele model, akin to the model of Rouzine and Coffin, and obtain estimates of *N_e_* by comparison with the data on *pro* and *env* regions employed by Rouzine and Coffin [15].

To validate our results against those of Rouzine and Coffin, we first perform simulations under the conditions they employ. We let *L* = 2 (two-locus/two-allele model). The fitness landscape is altered: the two single mutants have a fitness 1-*s* and the double mutant (1-*s*)^2^ relative to the wild-type. We let the founder sequence be the double mutant. Each cell is infected with a single virion (*M* = 1). Mutations occur with a probability *µ*. A cell infected by a provirus with fitness (1-*s*) is assumed to produce *P* = 20(1-*s*) progeny virions. Virions are then chosen randomly for infection. In each generation, the frequency of the least abundant haplotype is determined along with the frequencies of each of the alleles at the individual loci. The frequency of the least abundant haplotype is then averaged over those generations where the frequency of each allele is >25%. Several realizations are averaged to obtain the expected frequency of the least abundant haplotype (*lhf*).

**Figure 9:**
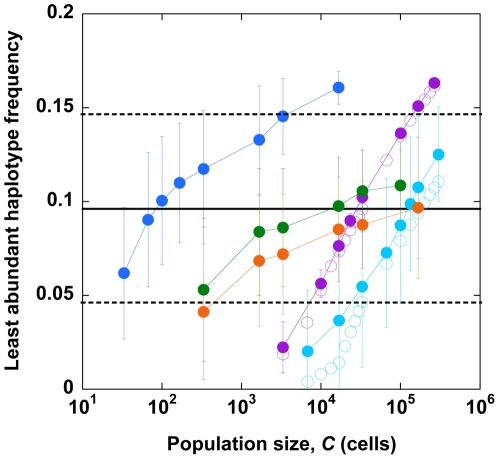
Estimation of *N_e_* using the linkage disequilibrium test. The frequency of the least abundant haplotype in a two-locus/two-allele model determined from our simulations (solid symbols) and by Rouzine and Coffin [15] (open symbols) as functions of the population size, *C*, assuming neutral evolution (purple), evolution with selection (cyan), and evolution with selection and recombination where the number of infections per cell is constant at 3 (blue), or follows a distribution determined from a viral dynamics model (see text) with *k_i_* = *k*_0_ (green) or *k_i_* = 0.7*k_i_*_-1_ (orange). Error bars represent standard deviations. Values of *C* at which predictions from simulations match experimental estimates [15] of the least abundant haplotype frequency (black line) yield *N_e_*. 95% confidence limits on the experimental data are also shown (dotted line).

Following Rouzine and Coffin, we perform simulations over a range of values of *µ* while keeping *C* fixed at 50 cells for neutral evolution (*s* = 0) and 5000 cells for evolution with selection (*s* = 0.1). For each *µ*, *Cµ/*(3×10^−5^) yields an equivalent population size corresponding to the HIV mutation rate of 3×10^−5^ substitutions per site per replication. *N_e_* is then obtained as that value of the equivalent population size at which predictions from simulations agree with experimental estimates of *lhf*. We find that our simulations are in excellent agreement with the results of Rouzine and Coffin both for neutral evolution and for evolution with selection (Fig. 9). These simulations yield *N_e_*∼10^5^ for evolution with selection, as deduced by Rouzine and Coffin. We note, as recognized by Rouzine and Coffin, that the mean experimental *lhf*≈0.09 yields *N_e_*∼10^5^, whereas the 95% confidence limit on the experimental data extends up to *lhf*≈0.14. Consequently, *N_e_*∼10^5^ is a lower bound and *N_e_* may even be ∼10^6^.

Recombination can increase *lhf* by inducing the association of mutations and therefore lower *N_e_*. We therefore include multiple infections of cells and recombination next in our simulations. We first let each cell be infected with *M* = 3 virions. In addition to mutation, recombination occurs at *ρl* crossovers per replication, where *l* = 71 nucleotides is the mean separation between the two loci [15], and *ρ = *8.3×10^−4^ crossovers per position per replication is the recombination rate. A cell infected by proviruses with mean fitness 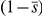 is assumed to produce *P* = 20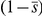 progeny virions. We perform simulations with a fixed *µ* = 3×10^−5^ substitutions per site per replication over a range of values of *C* and estimate *N_e_* as that value of *C* at which predictions agree with experimental estimates of *lhf*. We find that now *N_e_*∼10^2^–10^4^ (corresponding to the 95% confidence interval on *lhf*) (Fig. 9), consistent with our estimates of *N_e_* above (Fig. 3).

Next, we let *M* follow a distribution determined by a viral dynamics model (Methods). Here, we first assume that *k_i_*, the rate constant of the infection of a cell already infected with *i* proviruses, is equal to *k*_0_, the rate constant of the infection of uninfected cells, so that ∼70% of the cells are singly infected, ∼21% are doubly infected, and so on. Then, we find that *N_e_*∼10^4^ cells, higher than the estimate with *M* = 3 above. Next, following earlier studies [31], [33], we let *k_i_* = 0.7*k_i-_*_1_, which reduces the frequency of multiple infections even further, so that ∼77% of the cells are singly infected, ∼19% are doubly infected, and so on. With this distribution, we find *N_e_*∼10^5^, consistent with the estimate of Rouzine and Coffin. Note that when we ignore multiple infections of cells (*M* = 1), our simulations agree with those of Rouzine and Coffin. Thus greater the prevalence of multiple infections of cells and recombination, smaller is the estimate of *N_e_* obtained by the linkage disequilibrium test employed by Rouzine and Coffin.

## Discussion

The widely varying prevalent estimates of *N_e_* have been the subject of an ongoing debate [7] and have confounded descriptions of HIV-1 evolution, disease progression, and outcomes of therapy. We perform detailed bit-string simulations that closely mimic the evolution of HIV-1 diversity and divergence in patients. With parameter values representative of HIV-1 infection *in vivo*, our simulations provide good fits to longitudinal data of viral diversity and divergence over several years from nine patients [36] and yield estimates of *N_e_*. We find that *N_e_*∼10^3^–10^4^, substantially smaller than the inverse mutation rate of HIV-1, implying the predominance of stochastic forces underlying HIV-1 evolution *in vivo*. The small value of *N_e_* we estimate appears robust to variations in the frequency of multiple infections of cells and the fitness landscape.

The best-fit values of the viral generation time, τ, we obtain are in good agreement with prevalent estimates. We find that for the nine patients we consider the mean τ is 1.1–1.2 days (range 0.7–1.8 days). Previous studies estimate τ to be ∼1.2 days (range 0.65–2.97 days) using a coalescent approach [10], in the range 0.73–2.43 days using a pseudo-maximum likelihood approach [11], and ∼2 days from viral dynamics modeling [54], [55].

We find remarkably that *N_e_* and τ are correlated with disease progression. Smaller values of *N_e_* and τ correspond to smaller disease progression times, i.e., the time for the CD4^+^ T cell count to drop below 200 copies/µL, and thus to faster disease progression. A small τ implies fast viral replication and hence rapid disease progression. The origins of the correlation between *N_e_* and the progression time remain to be elucidated. Nonetheless, the strong correlations between *N_e_* and τ and the progression time imply that estimation of *N_e_* or τ, either through data of viral diversity and divergence as employed here or through independent techniques [10], [11], [54], [55], may allow *a priori* estimation of the disease progression time. Whether the correlations we observe are applicable over a larger number of patients remains to be ascertained.

Our simulations present an explanation of the wide variation in the prevalent estimates of *N_e_*. Whereas most studies estimate *N_e_*∼10^2^–10^4^ [3], [9]–[13], the study by Rouzine and Coffin obtains *N_e_*>10^5^ [15]. Previously, the large latter estimate has been suggested to arise from a bias introduced by restriction to data on polymorphic loci [13]: Rouzine and Coffin only consider loci where each allele is present in frequencies between 25% and 75%. Kouyos et al. [7] point out, however, that Rouzine and Coffin also restrict their simulations to similar polymorphic loci, thus eliminating any bias. Our simulations indicate that inclusion of multiple infections and recombination in the model of Rouzine and Coffin lowers *N_e_*. The multiplicative fitness profile employed in the latter model corresponds to vanishing epistasis. Thus, at any population size, random genetic drift produces negative linkage disequilibrium according to the Hill-Robertson effect [50]. Recombination lowers the absolute value of this linkage disequilibrium and results in an increase in viral diversity [21], [22], [32]. In effect, the frequency of the least abundant haplotype increases at any population size and results in lower *N_e_*. Thus, as the frequency of multiple infections increased from ∼80% cells being singly infected (*M* drawn from a distribution based on a viral dynamics model) to all cells triply infected (*M* = 3), *N_e_* decreased from ∼10^5^, in agreement with Rouzine and Coffin, to ∼10^2^ (Fig. 9).

We note that with the same frequency of multiple infections (*M* drawn from a distribution based on a viral dynamics model) and recombination, our simulations with the multi-locus model (*L* = 100) and comparisons with patient data of viral diversity yield *N_e_*∼10^4^ (Figs. 7 and 8), smaller than the value obtained by Rouzine and Coffin. We employ the same simulations to predict viral diversity and divergence (*d_G_* and *d_S_*) as well as the frequency of the least abundant haplotype (*lhf*), the latter quantity employed by Rouzine and Coffin. Thus, the difference in the resulting estimates of *N_e_* may be due to variations in model parameter values or due to the different calibration quantities employed. The differences in the model parameters we employ are in the genome length *L*, the fitness penalty *s*, and the virion production rate, *P*. Model predictions are weakly sensitive to variations in *P* (not shown). We employ *L* = 100 and *s* = 0.001 in our simulations of *d_G_* and *d_S_* and *L* = 2 and *s* = 0.1 for predicting *lhf*. The parameter values are chosen to match experimental observations. For instance, with *s* = 0.1, our simulations are unable to capture experimental observations of *d_G_* and *d_S_* (see Fig. 8). It is thus possible that mutations at the sites considered by Rouzine and Coffin may correspond to *s* = 0.1, whereas those in the patient data of Shankarappa et al. may be described by *s* = 0.001. If the latter possibility were true, then the difference in the estimate of *N_e_* may be attributed to the different calibration quantities employed; *lhf* may tend to yield higher values of *N_e_* than *d_G_* and *d_S_* with the present model of HIV-1 evolution.

Several factors may underlie the small values of *N_e_* we estimate compared to the census population of HIV-1 *in vivo*, *e.g.*, bottlenecks introduced by the immune system [3], [7], [9]–[13], asynchronous infections of cells [56], pseudohitchhiking [57], metapopulation structure [58], and variations in the progeny number across cells, the effects of which are yet to be fully elucidated.

**Figure 10:**
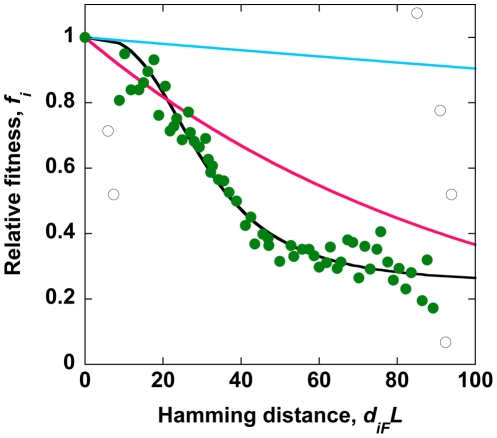
Fitness landscape. The relative fitness, *f_i_*, of genomes as a function of their Hamming distances from the fittest sequence, *d_iF_L*, obtained from experimental observations [37] (symbols) modified to account for the ratio of synonymous and non-synonymous substitutions (Methods) and predicted (black line) by the equation 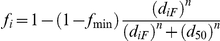, with the best-fit parameters *f*_min_ = 0.24, *d*_50_ *L* = 30, and *n* = 3 obtained upon ignoring outliers (open symbols). Multiplicative fitness landscapes, 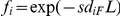, with *s* = 0.001 (cyan) and 0.01 (pink) are also shown.

Our study has limitations. First, the fitness landscape employed in our simulations (Fig. 10) has been determined for the reverse transcriptase and protease regions of HIV-1, whereas our simulations concern the *env* region. The fitness landscape has been determined experimentally by considering interactions between large numbers of mutations (up to ∼100) [37], and is thus the most detailed description of HIV-1 fitness available. Remarkably, the landscape indicates that the average fitness of genomes depends on the number of mutations (Hamming distance) and not on specific mutations, emphasizing the general nature of the fitness interactions. Thus, although individual mutations and fitness interactions between specific sites may be different for different regions of HIV-1, the generic interactions between loci are expected to be similar and captured by the experimental fitness landscape, allowing us to employ the landscape to describe the evolution of the *env* region of HIV-1.

Second, in some of the nine patients we consider, viral diversity rises to a peak and then drops to a plateau, whereas our simulations yield best-fits that predict a monotonic rise of the diversity to the plateau (Fig. 3). Our simulations do predict the non-monotonic evolution of diversity as the result of an interplay between mutation and fitness selection but only over certain ranges of mutation rates and fitness penalties (*s* = 0.01 in Fig. 8; also see [32]). For the parameter values that yield the best-fits to the patient data, our simulations predict a monotonic increase of diversity. The non-monotonic evolution of diversity could also be the result of HIV mediated collapse of the immune system, *i.e.,* immune relaxation [59], which our simulations ignore.

Nonetheless, our simulations incorporate the key evolutionary forces that govern HIV-1 diversification *in vivo* and describe quantitatively experimental observations of the time-evolution of viral diversity and divergence in patients over extended durations. Our simulations may thus be applied to predict determinants of disease progression and outcomes of therapy that are functions of viral diversity and divergence, such as the prevalence of drug-resistant strains prior to the onset of therapy, the role of recombination on the development of drug resistance, and the time of emergence and growth of drug-resistant and immune escape mutants [7], [22], [25], [33], [34], [60], which have remained difficult to determine experimentally.

The predominance of stochastic forces predicted by our estimates of *N_e_* has further implications for describing the within-host evolution of HIV-1 [7]. For instance, variations in disease progression may arise not only from host-genetic factors [4], [5], [61] but also from stochastic effects. A recent study attributed 15% of the inter-patient variation in the viral load in the chronic asymptomatic phase of infection to polymorphisms in a few host genes [61]. It would be of interest to identify the origins of the remaining 85% of the variation. Stochastic viral evolution implies that not all of the inter-patient variations may be attributable to variations in host-genetic factors. For instance, two individuals with identical genetic makeup may have distinct set point viral loads because viral genomes with different fitness may get fixed in the two patients by sheer chance. Accounting for stochastic variations in markers of disease state, for which our simulations present a framework, would lead to more robust associations between host-genetic factors and disease progression and facilitate more accurate identification of central players in HIV-1 pathogenesis.

## Methods

### Simulations of the within-host genomic diversification of HIV

We consider a fixed population, *C*, of uninfected cells exposed to a pool of *V* virions. Each virion consists of two RNA genomes represented by bit-strings of length *L* each. Infection begins with a pool of identical homozygous virions. We generate a sequence *F* of *L* nucleotides chosen randomly from A, G, C, and U. We let *F* be the fittest sequence and assign it the relative fitness of 1. We next generate the founder sequence, Θ, by mutating *F* in a fraction ξ of positions, chosen randomly. The founder sequence constitutes all the viral genomes in the initial viral pool [39].

We let infections occur in discrete generations. In any generation, each cell is infected by *M* virions drawn from the viral pool. A virion is chosen for infection with a probability equal to its relative fitness. Following infection, reverse transcription converts the viral RNA in each virion to proviral DNA of length *L*. Here, mutation and recombination introduce genomic variations. Mutations occur at *µ* substitutions per site per replication and recombination occurs at ρ crossovers per site per replication. The proviral DNA are then transcribed into viral RNA, which are assorted randomly into pairs and released as new virions. Each cell produces *P* progeny virions. The progeny virions form the viral pool for infection of the next generation of uninfected cells.

In each generation, we compute the average diversity, 
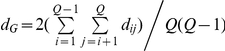
, of the *Q* proviruses present in that generation and their average divergence from the founder sequence, 
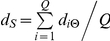
, where *d_ij_* is the Hamming distance per position between genomes *i* and *j*, and Θ represents the founder sequence. The Hamming distance between two genomes is the number of positions at which the two genomes differ. We also compute the average fitness of the *V* virions in each generation, 
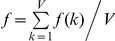
. The fitness *f*(*k*) of virion *k* containing genomes *i* and *j* is assumed to be the average (*f_i_*+*f_j_*)*/*2, where *f_i_* is the fitness of genome *i* (see below).

Several realizations of the infection process are averaged to obtain the expected evolution of *d_G_*, *d_S_* and *f*. The simulations are implemented using a computer program written in C++.

### Fitness landscape

For selection, we employ the recently determined experimental fitness landscape for HIV-1, which quantifies the fitness of a genome, *f_i_*, as a function of the Hamming distance between the amino acid sequence of that genome and that of the fittest genome, *F* [37]. The average ratio of non-synonymous to synonymous substitution rates in the *env* gene for the patient data of our interest is estimated to be ∼2.16 (Table 2) [14]. Consequently, we assume that a Hamming distance of 2.16 between amino acid sequences corresponds on average to a Hamming distance of 3.16 between nucleotide sequences. The resulting experimental fitness landscape in terms of the Hamming distance per position between nucleotide sequences is shown in Fig. 10. The latter landscape is well described by 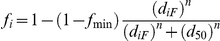, where *f_i_* is the relative fitness of genome *i* that has a normalized Hamming distance *d_iF_* from the fittest genome *F*, *f*_min_ = 0.24 is the minimum fitness of sequences attained at arbitrarily large absolute Hamming distances from *F*, *d_50_L* = 30 is that Hamming distance from *F* at which *f_i_* = 0.5(1+*f_min_*), *i.e.*, the average of the fittest and the least fit sequences, and *n* = 3 is analogous to the Hill coefficient (Fig. 10). We also perform simulations with a multiplicative fitness landscape, where 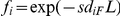 with *s* the fitness penalty per mutation.

**Table 2:** Estimates of synonymous and non-synonymous substitution rates.

Patient	Synonymous substitution rate (×10^−4^ per site per month)	Non-synonymous substitution rate (×10^−4^ per site per month)
1	3.5	5.4
2	3.1	8.7
3	3.7	14.1
5	7.9	8.5
6	4.5	5.2
7	2.2	9.5
8	3.7	9.2
9	1.6	5.3
**Mean**	**3.8**	**8.2**

The rates of synonymous and non-synonymous substitutions estimated by Lemey et al. [14] in the patients we consider (except Patient 11) from seroconversion until the CD4^+^ T cell count dropped to 200 cells/µL (mean 7 years). The ratio of the mean non-synonymous and synonymous substitution rates is 2.16.

### Frequency of multiple infections of cells from viral dynamics

To estimate the frequency of multiple infections of cells from viral dynamics, we consider the following model:
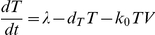


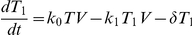


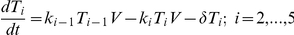


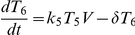


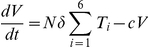



Here, uninfected CD4^+^ cells, *T*, are produced from the thymus at the rate *λ*, die with first order rate constant *d_T_*, and are lost due to infection by free virions with the second order rate constant *k*_0_. The latter infections produce singly infected cells, *T*_1_, which in turn are lost by further infections with the second order rate constant *k*_1_, or by death at the rate *δ*. Similarly, cells with two, three, etc. infections (*T_i_*, *i* = 2,3,…), are produced by successive infections. We restrict our model to a maximum of 6 infections per cell. (Including more infections does not alter our results significantly.) All infected cells produce virions at the rate *Nδ*, where *N* is the viral burst size. Virions are cleared at the rate *c*.

We solve these equations using parameter values representative of HIV-1 infection *in vivo* [55], [62]. We let *λ* = 10^5^ cells/ml/day, *d_T_* = 0.1/day, *k*_0_ = 2.4×10^−8^ ml/day δ = 1/day, *N* = 10^3^ virions/cell, and *c* = 23/day. The rates of infection of infected cells, *k_i_*, are not known. Virus-induced CD4 down-modulation [63], [64] would lower *k_i_* with increasing *i*. We have developed models previously that account for the continuous decrease of the susceptibility of cells owing to CD4 down-modulation following viral infection [31], [65]. A simplification of the latter models allowing up to two infections per cell found that *k*_1_ = 0.7*k*_0_ captured *in vitro* observations of the frequencies of coinfection quantitatively [31]. The cells employed in the latter *in vitro* experiments were not highly susceptible to infection. Other experiments found that coinfections occur more frequently than expected from random, independent infection events [66], [67], implying that *k*_1_>*k*_0_. Cell-to-cell transmission could also result in a high frequency of multiply infected cells [66], [68], [69]. Here, we therefore employ either *k_i_* = *k*_0_ or *k_i_* = 0.7*k_i-_*_1_ for all *i*. (Note that *M* = 3 corresponds to *k*_1_>*k*_0_.)

At long-times following the onset of infection, the above equations predict that infection reaches a steady state. The steady state populations of *T_i_* yield a distribution of the frequency of multiple infections of cells. We find at that steady state that ∼70% of the infected cells are singly infected, ∼21% are doubly infected, ∼6% are triply infected, ∼2% are quadruply infected, ∼0.5% are quintuply infected, and ∼0.2% are hextuply infected when *k_i_* = *k*_0_. Whereas, when *k_i_* = 0.7*k_i-_*_1_, at steady state ∼77% of the infected cells are singly infected, ∼19% are doubly infected, ∼3.4% are triply infected, ∼0.5% are quadruply infected, ∼0.04% are quintuply infected, and ∼0.002% are hextuply infected.
